# Detection of Natural Resistance-Associated Substitutions by Ion Semiconductor Technology in HCV1b Positive, Direct-Acting Antiviral Agents-Naïve Patients

**DOI:** 10.3390/ijms17091416

**Published:** 2016-08-27

**Authors:** Nadia Marascio, Grazia Pavia, Alessio Strazzulla, Tim Dierckx, Lize Cuypers, Bram Vrancken, Giorgio Settimo Barreca, Teresa Mirante, Donatella Malanga, Duarte Mendes Oliveira, Anne-Mieke Vandamme, Carlo Torti, Maria Carla Liberto, Alfredo Focà, the SINERGIE-UMG Study Group

**Affiliations:** 1 Department of Health Sciences, Institute of Microbiology, School of Medicine, University of Magna Graecia, Viale Europa, Germaneto, 88100 Catanzaro, Italy; grazia_pavia@libero.it (G.P.); gbarreca@unicz.it (G.S.B.); mliberto@unicz.it (M.C.L.); alfredofoca@gmail.com (A.F.); 2 Katholieke Universiteit (KU) Leuven–University of Leuven, Department of Microbiology and Immunology, Rega Institute for Medical Research, Clinical and Epidemiological Virology, 3000 Leuven, Belgium; tim.dierckx@kuleuven.be (T.D.); lize.cuypers@kuleuven.be (L.C.); bram.vrancken@kuleuven.be (B.V.); annemie.vandamme@uzleuven.be (A.-M.V.); 3 Department of Medical and Surgical Sciences, Unit of Infectious and Tropical Diseases, School of Medicine, University of Magna Graecia, Viale Europa, Germaneto, 88100 Catanzaro, Italy; alessiostrazzulla@yahoo.it (A.S.); torti@unicz.it (C.T.); 4 Centro di Servizio Interdipartimentale (CIS)-Genomica funzionale e Patologia Molecolare, University of Magna Graecia, Viale Europa, Germaneto, 88100 Catanzaro, Italy; teresa87@tiscalinet.it; 5 Department of Experimental and Clinical Medicine, University of Magna Graecia, Viale Europa, Germaneto, 88100 Catanzaro, Italy; malanga@unicz.it (D.M.); oliveira@unicz.it (D.M.O.); 6 Center for Global Health and Tropical Medicine, Institute for Hygiene and Tropical Medicine, University Nova de Lisboa, Rua da Junqueira 100, 1349-008 Lisbon, Portugal

**Keywords:** hepatitis C virus 1b, resistance-associated substitutions, direct-acting antiviral agents, deep-sequencing

## Abstract

Naturally occurring resistance-associated substitutions (RASs) can negatively impact the response to direct-acting antivirals (DAAs) agents-based therapies for hepatitis C virus (HCV) infection. Herein, we set out to characterize the RASs in the HCV1b genome from serum samples of DAA-naïve patients in the context of the SINERGIE (South Italian Network for Rational Guidelines and International Epidemiology, 2014) project. We deep-sequenced the NS3/4A protease region of the viral population using the Ion Torrent Personal Genome Machine, and patient-specific majority rule consensus sequence summaries were constructed with a combination of freely available next generation sequencing data analysis software. We detected NS3/4A protease major and minor variants associated with resistance to boceprevir (V36L), telaprevir (V36L, I132V), simeprevir (V36L), and grazoprevir (V36L, V170I). Furthermore, we sequenced part of HCV NS5B polymerase using Sanger-sequencing and detected a natural RAS for dasabuvir (C316N). This mutation could be important for treatment strategies in cases of previous therapy failure.

## 1. Introduction

Treatment of hepatitis C virus (HCV) infection has significantly improved in the past few years after the introduction of direct-acting antivirals (DAAs) agents [1]. While patients treated with pegylated interferon-α (pegIFN-α) plus ribavirin (RBV) reached sustained virological response (SVR) in only 42% of cases [2], clinical trials have demonstrated that combinations including a first-generation DAA and pegIFN-α + RBV are effective in obtaining a SVR in up to 75% of patients infected with HCV genotype 1 (1a and 1b subtypes) [3]. Lately, new interferon-free regimens are able to eradicate HCV infection in more than 90% of patients after 12 weeks of treatment, irrespective of the HCV genotype [4].

However, the targets of these DAAs are viral proteins, and due to the rapid evolution of the virus, viral variants resistant to drugs can emerge and lead to therapy failure [5,6]. For this reason, it is sometimes advised to consider the presence of resistance-associated substitutions (RASs) at therapy initiation, and now importantly also at virological failure, to guide drug selection and for the rational management of HCV infection [1,7,8,9].

In order to prescribe the appropriate treatment with regards to the drug type and length of treatment, determining the correct HCV subtype is crucial [1]. Likewise, the genetic background of different subtypes is also important for the interpretation of RASs [7]. In clinical practice, the infecting subtype is determined by a reverse hybridization line probe assay (LiPA) that distinguishes subtypes based on the heterogeneity in the 5′UTR and core regions, with a misclassification rate of around 11% [10]. Indeed, it has become clear that more accurate subtyping results can be obtained through the analysis of more diverse regions, such as the NS5B gene [11,12,13]. Recently, Di Maio et al. [14] reported that HCV NS3 sequencing, followed by phylogenetic analysis, also improves the specificity and sensitivity of subtype assignments.

The large diversity of HCV [15] complicates the bulk sequencing of viral populations with Sanger-sequencing. Furthermore, population sequencing can only reliably detect variants that make up around 20% of the viral population [16], which makes this a suboptimal approach for assessing the impact of lower-frequency RASs on therapy outcome [9]. These limitations make massively parallel sequencing methods an attractive alternative for the study of complex virus populations [17]. Indeed, next-generation sequencing (NGS) has been successfully applied in a wide range of HCV analyses, including characterization of virus transmission [18] and positions of mutation (hot spot) in NS3/4A protease [19].

The vast amount of data produced by deep sequencing platforms, each with its particular characteristics, stimulated bioinformatics developments to efficiently translate the raw sequence data into interpretable information [20,21]. The choice of NGS platform and post-processing largely depend on the research purpose. The short read platforms (e.g., the Illumina^®^ sequencers) are currently the most widely used, but the advantages of longer read lengths in reads assembly and the preservation of mutational linkage over longer distances [22,23,24] ensure a place for longer read technologies, such as the Ion Torrent platforms for virus sequencing. As a case in point, the Ion Personal Genome Machine (PGM) Sequencer (Life Technologies, Carlsbad, CA, USA) has recently been used to detect low-level drug resistance variants in HCV and HIV *quasispecies* [25,26,27,28,29].

Herein, we used the PGM sequencer in concert with a number of freely available analysis software packages [30,31,32,33,34] to generate a baseline resistance profile for eight samples from DAA-naïve patients chronically infected with HCV1b, targeting NS3 protease. Also, the NS5B Sanger sequences, used to genotype/subtype virus, were screened for RASs to polymerase inhibitors.

## 2. Results

**Figure 1:**
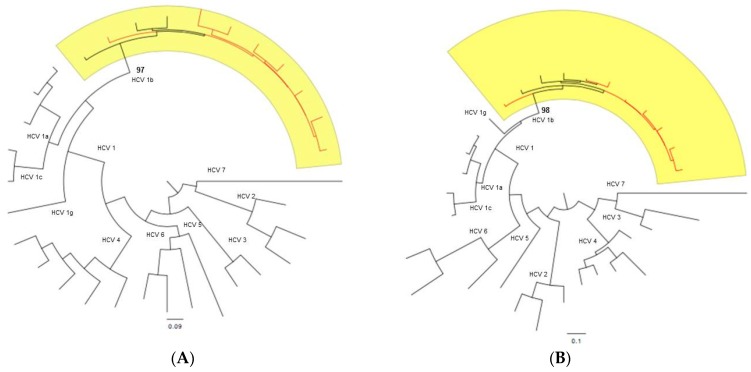
RAxML phylogenetic trees were estimated using 24 hepatitis C virus (HCV) reference sequences (black) downloaded from Los Alamos HCV Sequence Database and 8 HCV isolates (red) in this study for NS3 (**A**) and NS5B (**B**) regions, respectively. The reliability of the phylogenetic clustering was evaluated using bootstrap analysis with 1000 replicates. Bootstrap support values are only shown for the HCV1b clades (yellow area). The scale bars at the bottom of the figure represent genetic distance.

The Versant HCV genotype 2.0 assay classified HCV isolates from all patients as HCV subtype 1b, except for one patient whose genotype was characterized as a mixed genotype 1b/4 (Table 1). In contrast, all samples were classified as genotype 1b by both the Oxford and COMET subtyping tools, and by phylogenetic analysis of the NS3 (Figure 1A) and NS5B (Figure 1B) regions.

Seven patients were previously treated with pegIFN-α/RBV and were classified as partial responders (3/7) or relapsers (4/7). The eighth patient was naïve to any prior HCV treatment. The median age of the patients was 55 years (interquartile range (IQR): 46.8–66.5), and six out of eight patients were males. Concerning transmission route, surgery was the most frequently reported risk factor (Table 1).

HCV RNA levels during follow-up (scheduled according to official guidelines EASL 2015) [1] and adverse events for each patient are listed in Table 2. The median baseline RNA viral load was 2,130,000 IU/mL. At week 4 of treatment, no HCV RNA was detected for 3/8 samples (HCV04, HCV08, HCV20 patients) while for 4 samples a viremia of <15 IU/mL was measured, and patient HCV21, who was treated with boceprevir (BOC), had an HCV RNA level of 11,900 IU/mL. Viremia was <15 IU/mL at 8 weeks in patient HCV21. All patients had undetectable RNA from week 12 to the end of therapy. Patients were asked to report any adverse events; three patients (HCV09, HCV17, HCV21) reported anemia and one patient (HCV20) reported anemia and neutropenia. The initial liver stiffness was tested by FibroScan^®^ (Table 1) for its association with SVR and the occurrence of adverse events (Table 2). In particular, among our patients: 5/8 (HCV04, HCV06, HCV08, HCV09, HCV19) were classified as Metavir F0−F1, 1 patient (HCV17) was classified as F3, and 2/8 (HCV20, HCV21) were classified as F4 (Table 1).

We identified several nonsynonymous substitutions in the majority rule NS3 consensus sequences (Table 3), and in minor viral populations. Substitution _NS3_V36L—conferring resistance to BOC and possibly to telaprevir (TVR) or simeprevir (SMV) [5] or grazoprevir (GZR) [36]—was detected in a treatment-naïve patient’s (HCV17) HCV isolate. We also identified substitution _NS3_I132V [29], which is associated with possible resistance to TVR, in 4 patients: HCV04, HCV06, HCV08, HCV19. The _NS3_V170I GZR RAS was found in HCV isolate from patient HCV20 [37] (Table 3). However, for minor substitutions in the NS3 region, nucleotide substitutions were detected at amino acid (AA) positions 55 (10.9%) and 132 (0.05%) in isolates from HCV17 and HCV21 patients, respectively, but these changes did not modify the corresponding AA (i.e., synonymous substitutions). The _NS5B_C316N mutation associated with resistance to the NS5B polymerase inhibitor, dasabuvir (DSV) [38], was found in two patients (HCV06, HCV19), who also harbored _NS3_I132V and _NS5B_C316N (Table 3).

We also found genotype 1b polymorphisms [39], _NS5B_V338A and _NS3_D30E + _NS3_I170V, in all HCV isolates (Table 3). A mutation analysis for the last generation DAAs showed that none of the isolates harbor RASs for NS3/4A protease inhibitor paritaprevir or NS5B polymerase inhibitor sofosbuvir [5] (Table 3).

## 3. Discussion

In this work we capitalized on the sequence output of the Ion Torrent PGM Sequencer to generate a baseline NS3 resistance profile of eight DAA-naïve patients infected by HCV1b subtype. The NS5B coding region of the same isolates was sequenced with conventional Sanger technology for genotyping purposes, and was also screened for RASs to polymerase inhibitors.

We performed bulk sequencing of virus populations and were able to construct a consensus representation of the intra-host viral population so that secondary variants could also be detected. However, the threshold for clinical relevance of RASs in population sequencing is set at 15%–25% [40]. This contrasts sharply with the excellent in-depth view of the viral population composition that can be achieved with NGS methods, even though replicate analysis is advised to average the effects of random variations in the sample pre-processing procedures [23,41]. We have applied this technique for mutation analysis to a sample of clinical isolates. Although the predictive potential of baseline resistance substitutions remains controversial, RASs have been associated with reduced response to DAA regimens in several studies [5,6]. Using deep sequencing with PGM, we conducted a thorough assessment on HCV NS3 protease major and minor variants in HCV1b isolates from samples of chronic DAA-naïve patients; samples were gathered prior to the telaprevir- or boceprevir-based triple therapy. In addition, once NS5B sequences were obtained by conventional Sanger-sequencing method for subtyping analysis, we also looked for RASs to polymerase nucleotide inhibitors within the dominant viral population [42]. A potential useful application of deep sequencing is that this technique could detect minor variants, whose impact on clinical response to treatment is significant.

We found a dual resistance profile, _NS3_I132V and _NS5B_C316N, against TVR (a protease inhibitor) and DSV (a polymerase inhibitor), respectively, in two patients (Table 3). This finding underlines the value of a wider genomic view on resistance testing when multiple viral proteins are targeted [39]. The fact that all patients (including those with RASs) reached SVR with the TVR- or BOC-based triple therapy seems to suggest that other factors, besides the presence of RASs, contribute to the eventual treatment response. The present study adds to the available information regarding the prevalence of baseline HCV RASs and polymorphisms. Furthermore, we recognize that the studied drug combinations remain acceptable in selected patients for whom the new DAAs regimens are not available [1] and further studies involving patients treated with current regimens are needed. Lastly, the sequenced NS5B region, used for subtyping analysis, allowed us to identify _NS5B_C316N RAS even though a number of important AA positions for resistance against NS5B non-nucleoside inhibitors (i.e., 368, 411, 414, 448, 553, 554, 556) were not amplified by the used primers [43,44].

In the NS3 region, we found two RASs, _NS3_V36L [45] and _NS3_I132V [29], in isolates from one patient and four patients, respectively. The _NS3_V36L variant carried by patient HCV17 confers resistance to BOC and possibly to TVR or SMV and is reported to be present in 0.5% of all previously studied worldwide HCV1b strains [46]. An Italian study of 326 HCV-infected patients treated with TVR/BOC triple therapy, showed this _NS3_V36L variant in only two HCV1b patients with SVR [14], in line with our observations. The two isolates from HCV06 and HCV19 patients carried mutations for TVR (_NS3_I132V) and DSV (_NS5B_C316N). In phase 3 clinical trials, the _NS3_I132V was reported as RAS only for the HCV1a subtype [45], although _NS3_132V has recently been described as an HCV1b-resistant variant with a high prevalence at baseline [29]. In particular, _NS3_I132V variant is present at 0.5% and 73.7% in HCV1a versus HCV1b strains [46]. The _NS3_V170I GZR RAS, detected at baseline in patient HCV20, has also been reported at treatment failure during a randomized trial of HCV1b-infected patients [37].

As reported in the results section, in some cases (HCV isolates from patients HCV04, HCV06, HCV08, HCV17, and HCV19) RASs were detected but SVR was achieved. This may reflect an incomplete knowledge of the mutation patterns that have a clinical meaningfulness (i.e., predictive value for the risk of virological failure). Unfortunately, our sample size was too small to address these issues with an acceptable statistical power. Moreover, it is possible that host variability (including pharmacogenomical features, such as IL28B single nucleotide polymorphisms) may have favored virological response notwithstanding the presence of resistant virus *quasispecies*. Unfortunately, however, we did not include any pharmacogenomical evaluations and, again, our sample size was limited. Further studies are needed in this respect.

High-throughput deep sequencing allowed us to determine minor nucleotide variations in the viral population of each patient. The analysis highlighted nucleotide mutations in hot spot positions, ranging 0.05%–10.9%, that were not associated with HCV resistance to DAAs.

Our combination of the PGM and software packages was able to identify low frequency RASs in the viral *quasispecies* population, which could provide important insights when the methodology used in this study is applied in a larger cohort of patients.

The NS5B variant C316N, known to confer resistance to non-nucleoside inhibitor DSV, is observed in 31.4% of the worldwide HCV1b strains [46], and assessment of this mutation could be important for the treatment strategy in case of TVR failure. Indeed, DSV is approved by the FDA (Food and Drug Administration) in combination with other DAA agents in interferon-free regimens for HCV1, achieving high cure rates with few adverse effects [42]. Therefore, in case of TVR treatment failure, DSV could be contraindicated in the presence of the _NS5B_C316N variant. Furthermore, we found a substantial number of polymorphisms (Table 3) in the NS3 protease and NS5B polymerase regions, which could be characterized as RASs in the near future. These substitutions could prove to be useful for drug resistance studies [46], and our report adds to the knowledge regarding their general prevalence in Italian HCV isolates. For instance, Chen et al. [9] described the _NS3_I170V variant, observed in 7/8 of our patients, as BOC RASs in HCV1a, HCV2, HCV3, HCV4 and HCV6 genotypes, while the Geno2pheno team describes them as mutations in a hot spot position not yet related to resistance against BOC in HCV1b strains. We also highlight the polymorphism _NS3_S122N found in a hot spot position of the NS3 protease region. Lontok et al. recently reported A/G/I/T substitutions in the same amino acid residue at the time of virological failure [5]. Analogous to what has been observed in HIV studies, AA substitutions could be crucial for future analyses and possible correlations with the clinical outcome [47].

## 4. Materials and Methods

### 4.1. Ethic Statement

The study was approved by the Ethical Committee (#2012.58.E; 19 June 2013) of the Mater Domini University Hospital of Catanzaro, Italy. Written informed consent was obtained from each patient in accordance with the principles of the Helsinki Declaration (World Medical Association General Assembly, Seoul, Korea, 59 October 2008).

### 4.2. Study Population

Eight DAA-naïve patients chronically infected with HCV subtype 1b, who were not co-infected with HIV or HBV, were recruited between January and December 2014 at the “Mater Domini” University Hospital of Catanzaro, Italy, as part of the SINERGIE (South Italian Network for Rational Guidelines and International Epidemiology) project [48]. We provide an overview of the patients’ clinical information in Table 1. After baseline sampling and evaluation of initial fibrosis stage, a triple therapy protocol (pegIFN-α and RBV supplemented with telaprevir or boceprevir) was started for each patient [1].

### 4.3. HCV RNA Viral Load Determination

HCV RNA viral load was determined with the Cobas AmpliPrep/Cobas TaqMan HCV test (Roche Diagnostics, Milan, Italy) (quantification range of 15 to 100 million IU/mL).

### 4.4. Liver Stiffness

Fibrosis stage was estimated by transient elastometry (FibroScan^®^), interpreted as follows: KPa ≤ 7.1 = F0−F1 (minimal fibrosis), 7.1 < KPa ≤ 9.5 = F2 (moderate fibrosis), 9.5 < KPa ≤ 14.5 = F3 (severe fibrosis), and KPa > 14.5 = F4 (cirrhosis) [35].

### 4.5. HCV NS3 Protease Deep Sequencing

**Figure 2:**
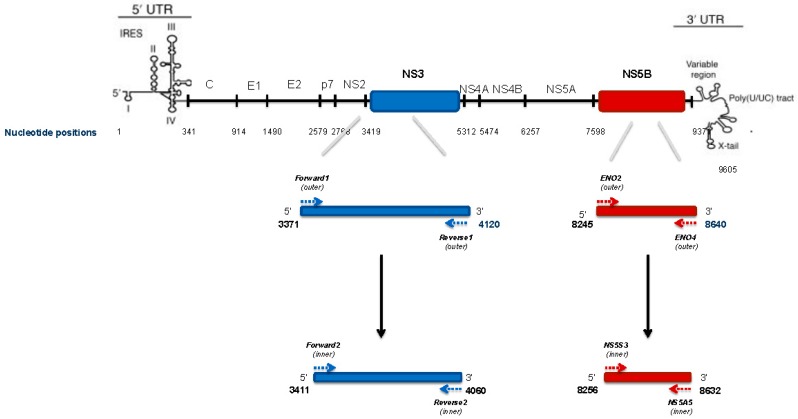
PCR primers positions and amplicons lenght for NS3 and NS5B HCV genomic regions, according to the HCV1b reference sequence (Con1 AJ238799).

Viral RNA was extracted from 140 µL serum using the QIAmp viral RNA extraction kit (Qiagen, Hilden, Germany) in accordance with the manufacturer’s protocol. RNA was reverse-transcribed using the High-Capacity cDNA Reverse Transcription Kits protocol (Applied Biosystems, Foster City, CA, USA). The 2X reverse transcription Master Mix (2 µL 10× RT Buffer, 0.8 µL 25× dNTP Mix (100 mM), 2 µL 10× RT Random Primers, 1 µL MultiScribe™ Reverse Transcriptase, 1 µL RNase Inhibitor and RNase-free water) was added at 10 µL RNA to make up a final volume of 20 µL. Synthesized cDNA was amplified by PCR technique using in-house-developed primers specific for NS3 genomic region (650 bp), covering all NS3/4A positions involved in drug resistance using GoTaq^®^ DNA Polymerase (Promega, Madison, WI, USA). The nested PCR amplification for both rounds was performed under the following conditions: 1.25 units of GoTaq^®^ DNA Polymerase, 50 mM KCl, 30 mM Tris-HCl, 1.5 mM Mg^2+^, 200 µM of each dNTP, 0.2 µM sense primer, 0.2 µM antisense primer. Six microliters of cDNA was used for the first round of PCR. NS3 primers were the following: Forward1 (outer) 5′-GGAGGGAGATACATCTGG-3′; Reverse1 (outer): 5′-GTTCAGGACAAGCACCTTAT-3′. Thermocycling conditions consisted in a denaturation step at 95 °C for 5 min, followed by 35 cycle at 95 °C for 30 s; 62 °C for 30 s; 72 °C for 1 min; a final elongation cycle of 7 min at 72 °C and finally a hold at 4 °C. Five microliters of the first round of PCR products was used for nested-PCR, using the same thermocycling conditions. NS3 nested primers were the following: Forward2 (inner) 5′-ACTCCTCGCGCCTATTACG-3′; Reverse2 (inner) 5′-TTAGTGCTCTTGCCGCTACC-3′ (Figure 2). In order to obtain enough material for sequencing, the second PCR was performed in duplicate. These PCR products were quantified by semi-fluidic electrophoresis (Agilent^®^ Bioanalyzer^®^, Santa Clara, CA, USA), using Agilent High Sensitivity DNA Kit and diluted to in order to obtain 100 ng of HCV cDNA in a final volume of 40 µL.

High-throughput sequencing was performed using the Ion Torrent Personal Genome Machine (PGM) Sequencer [49]. The adapter- and barcoded-ligated library was prepared according to manufacturer’s protocol using the Ion Xpress Plus Fragment Library kit (Life Technologies, Foster, CA, USA). Briefly, amplicons were enzymatically fragmented for 5 min, using Ion Shear Reagents. Fragments were then purified by Agencourt^®^ AMPure^®^ XP Reagent and the fragmentation pattern was analyzed by semi-fluidic electrophoresis (Agilent^®^ Bioanalyzer^®^, Santa Clara, CA, USA) Barcodes and adapters were ligated to the amplicons and library fragments of 300–400 nucleotides long were size selected using E-gel^®^ SizeSelect Agarose Gel (Life Technologies, Carlsbad, CA, USA). Emulsion PCR and Ion sphere particle (ISP) enrichment was performed by using Ion OneTouch 400 Template Kit (Life Technologies). The enrichment of template on Ion Spheres particles (ISPs) was quantified using Qubit System (Life Technologies) and loaded into a 314 chip, according to Ion PGM Hi-Q sequencing kit protocol.

### 4.6. HCV NS3 NGS Read Analysis

**Figure 3:**
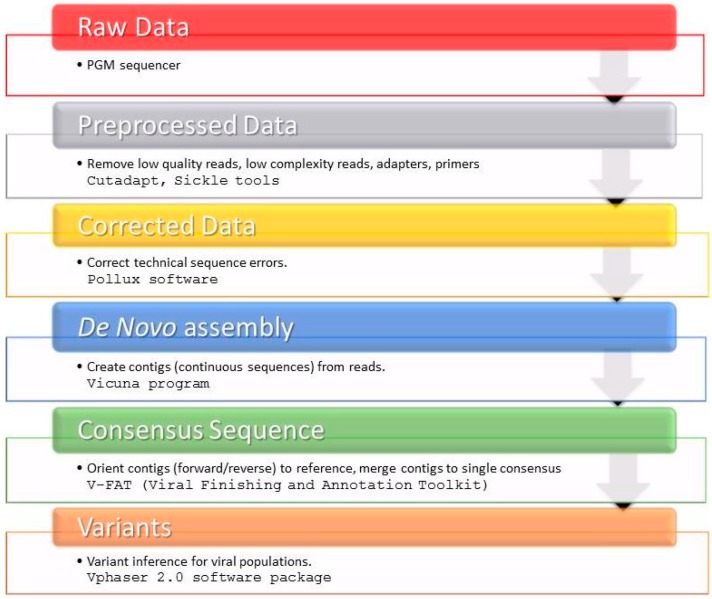
Workflow of sample-specific consensus sequence of the NS3 region generated by an in-house-developed script.

We provide an overview of the sequence read post-processing steps in Figure 3. Briefly, Cutadapt [31] and Sickle tools [30] were used to remove low quality reads, low complexity reads, adapters and primers from fastq raw sequence data. After quality control of raw data using FastQC (Available online: http://www.bioinformatics.babraham.ac.uk/projects/fastqc/) and pre-processing with the Pollux error correction software [32], a de novo assembly was performed using software packages VICUNA [33] and V-FAT [34]. The sample-specific majority rule consensus sequences were used to infer the variant frequencies with V-phaser 2 [50], and we only considered variants that had no strand bias (*p*-value ≤ 0.05).

### 4.7. Subtyping Analysis

The Versant HCV genotype v2.0 assay (LiPA, Siemens, Healthcare Diagnostic Inc., Tarrytown, NY, USA) was used for initial subtyping. We sought to confirm the subtype assignment by three methods using the NS3 majority rule consensus sequences (cfr. above) and the NS5B population consensus sequence. The NS5B region was amplified as previously described (Figure 2) [43,44] and sequenced by the traditional dideoxy chain termination method using ABI PRISM 3500 genetic analyzer (Applied Biosystems). These were submitted to the Oxford HCV Automated Subtyping Tool v.2.0 [51] and the COMET HCV typing tool [52], and were also used in a maximum likelihood phylogenetic inference incorporating reference sequences available from the Los Alamos National Laboratory HCV Sequence Database (Table S1) [53]. Sequences were aligned with Clustal W [54] and manually edited in MEGA v.5.2.2 [55]. Trees were estimated, inferring with the generalized time reversible (GTR) nucleotide substitution model with gamma (Г)-distribution by RAxML v.8.1.17 [56] and the reliability of phylogenetic clustering was evaluated using 1000 bootstrap replicates. Phylogenetic trees were visualized with FigTree v1.4.2 (Available online: http://tree.bio.ed.ac.uk/software/figtree/).

### 4.8. Genetic Variability Analysis

We compiled a list of positions known to possibly encode for RASs through a literature search [5,9,29] and with the Geno2pheno_[HCV]_ 0.92 tool [57] using the rules as updated on 21 October 2015. Additionally, the same amino acid (AA) substitutions, aligning generated sequences to HCV1b (accession number AJ238799) wild-type reference by MUSCLE [58], were confirmed. This resulted in the following list of NS3 AA positions: 36, 43, 54, 55, 80, 107, 122, 132, 155, 156, 168, 170, 174, 175; for NS5B we evaluated variation at positions 282, 316, 321.

### 4.9. Public Availability of the Sequencing Data

The newly generated NS3 and NS5B sequences were submitted to the SINERGIE database (available from ARCA (dbARCA)-Antiviral Response Cohort Analysis (Available online: https://www.dbarca.net/)) and to GenBank^®^ database [59]. All sequences can be retrieved from GenBank^®^ under accession numbers: KX373275-KX373290.

## 5. Conclusions

In conclusion, we found pretreatment RASs in 6/8 patients. In spite of the presence of RASs, all patients achieved SVR at 12 weeks under TVR or BOC plus pegIFN-α/RBV triple therapy regimens. However, further studies focusing on current treatments are necessary to establish the impact of RASs and other polymorphisms on treatment response. We have determined that the combination of the PGM Sequencer in conjunction with an analysis pipeline consisting of freely available software could be a useful method to analyze genetic variability in the NS3 coding region. Our workflow is readily extensible to a wider genome perspective, but requires benchmarking on simulated data sets and/or larger patient cohorts. This piece of evidence will be extended through implementation of an ongoing international collaboration.

## Figures and Tables

**Table 1:** Patients’ characteristics.

Patient ID	Gender	Age	LiPA Genotyping	Liver Stiffness (KPa) *	Risk Factors ^#^	Response Previous Therapy
HCV04	female	47	1b	6.0	Surgery and Cohabitation	Partial responder
HCV06	male	68	1b	6.5	Surgery and Cohabitation	Partial responder
HCV08	male	46	1b	6.0	Surgery	Relapser
HCV09	male	62	1b/4	6.1	Surgery and Cohabitation	Relapser
HCV17	female	69	1b	14.0	Surgery	Naïve
HCV19	male	45	1b	6.9	Surgery and Tattoo	Relapser
HCV20	male	51	1b	22.0	Surgery	Relapser
HCV21	male	66	1b	25.0	Not Available	Partial responder

* According to Castéra et al. [35], 2005, KPa ≤ 7.1 = F0−F1 (minimal fibrosis), 7.1 < KPa ≤ 9.5 = F2 (moderate fibrosis), 9.5 < KPa ≤ 14.5 = F3 (severe fibrosis), and KPa > 14.5 = F4 (cirrhosis); ^#^ Surgery and cohabitation with HCV-antibodies (Ab) positive individuals or tattoo are transmission risks reported by the same patient.

**Table 2:** Direct-acting antiviral (DAA) therapy, HCV RNA viral load, adverse events, and treatment response.

Patient ID	DAA Therapy *	HCV RNA (IU/mL)						Adverse Events	SVR 12
–	–	Baseline	4 Weeks	12 Weeks	24 Weeks	36 Weeks	48 Weeks	–	–
HCV04	TVR	7,410,000	TND	TND	TND	TND	TND	None	Yes
HCV06	TVR	1,580,000	<15	TND	TND	TND	TND	None	Yes
HCV08	TVR	2,090,000	TND	TND	TND	TND	TND	None	Yes
HCV09	TVR	7,930,000	<15	TND	TND	TND	TND	Anemia	Yes
HCV17	TVR	2,170,000	<15	TND	TND	TND	TND	Anemia	Yes
HCV19	TVR	4,290,000	<15	TND	TND	TND	TND	None	Yes
HCV20	TVR	1,520,000	TND	TND	TND	TND	TND	Anemia and Neutropenia	Yes
–	–	Baseline	4 Weeks	8 Weeks	12 Weeks	24 Weeks	36 Weeks	–	–
HCV21	BOC	1,030,000	11,900	<15	TND	TND	TND	Anemia	Yes

SVR = Sustained Virological Response; TND = Target Not Detected; * Patients HCV04, HCV06, HCV09, HCV17, HCV19 and HCV20 were treated with pegylated interferon-α plus ribavirin (PegIFN-α/RBV) + telaprevir (TVR) for 12 weeks, afterward with PegIFN-α/RBV for 36 weeks; Patient HCV08 was treated with PegIFN-α/RBV + TVR for 12 weeks, afterward with PegIFN-α/RBV for 12 weeks; Patient HCV21 was treated with PegIFN-α/RBV for 4 weeks, afterward with PegIFN-α/RBV + boceprevir (BOC) for 44 weeks.

**Table 3:** Nonsynonymous substitutions detected for NS3 and NS5B target region in HCV isolates from SVR patients.

Patient ID	NS3 Protease		NS5B Polymerase	
–	RASs	Polymorphisms	RASs	Polymorphisms
HCV04	I132V	S7A	none	V338A
		D30E		
		P86Q		
		M94L		
		V114I		
		S122N		
		I170V		
HCV06	I132V	L14F	C316N	R254K
		D30E	–	S300T
		V48I		V338A
		P86Q		–
		P89S		
		M94L		
		V114I		
		S122N		
		V150A		
		I170V		
HCV08	I132V	D30E	none	V338A
		T72I		–
		P86Q		
		M94L		
		V114I		
		V150A		
		I170V		
HCV09	none	D30E	none	L266M
		V48I		V338A
		T72I		–
		P89S		
		M94L		
		H110Y		
		S122N		
		V150A		
		I170V		
HCV17	V36L	L14I	none	A252V
		D30E		Q309R
		V48I		V338A
		S61A		–
		P86Q		
		A87S		
		M94L		
		V114I		
		V150A		
		I170V		
HCV19	I132V	L14F	C316N	Q309R
		D30E	–	S335N
		S61T		V338A
		P86Q		–
		M94L		
		V114I		
		V150A		
		I170V		
HCV20	V170I	D30E	none	Q309R
		S61A		S335N
		P86Q		V338A
		M94L		–
		V114I		
		V150A		
HCV21	none	L14F	none	V338A
		D30E		–
		V48I		
		I71V		
		T95A		
		V114I		
		V150A		
		I170V		

## Supplementary Materials

Supplementary materials can be found at www.mdpi.com/1422-0067/17/9/1416/s1.
