# Supplementary material for: Detection of Natural Resistance-Associated Substitutions by Ion Semiconductor Technology in HCV1b Positive, Direct-Acting Antiviral Agents-Naïve Patients
Source: Int J Mol Sci. 2016 Aug 27;17(9):1416. doi: 10.3390/ijms17091416 (PMC5037695; doi:10.3390/ijms17091416)
Supplement: Supplementary file 1 [file ijms-17-01416-s001.pdf]

# Supplementary Materials: Detection of Natural Resistance-Associated Substitutions by Ion Semiconductor Technology in HCV1b Positive, Direct-Acting Antiviral Agents-Naïve Patients

**Nadia Marascio, Grazia Pavia, Alessio Strazzulla, Tim Dierckx, Lize Cuypers, Bram Vrancken, Giorgio Settimo Barreca, Teresa Mirante, Donatella Malanga, Duarte Mendes Oliveira, Anne-Mieke Vandamme, Carlo Torti, Maria Carla Liberto, Alfredo Focà and the SINERGIE-UMG Study Group**

**Table S1.** Accession numbers of NS3 and NS5B reference sequences available in Los Alamos HCV Sequence Database.

| Accession Number | HCV Subtype | Genomic Region |
|---|---|---|
| M67463 | 1a | NS3 and NS5B |
| EF407419 | 1a | NS3 and NS5B |
| AF511950 | 1a | NS3 and NS5B |
| EF032892 | 1b | NS3 and NS5B |
| AY587016 | 1b | NS3 and NS5B |
| D11355 | 1b | NS3 and NS5B |
| D14853 | 1c | NS3 and NS5B |
| AY051292 | 1c | NS3 and NS5B |
| AM910652 | 1g | NS3 and NS5B |
| AY746460 | 2a | NS3 and NS5B |
| D10988 | 2b | NS3 and NS5B |
| D50409 | 2c | NS3 and NS5B |
| X76918 | 3a | NS3 and NS5B |
| D49374 | 3b | NS3 and NS5B |
| Y11604 | 4a | NS3 and NS5B |
| FJ462435 | 4b | NS3 and NS5B |
| FJ462436 | 4c | NS3 and NS5B |
| FJ462437 | 4d | NS3 and NS5B |
| EF589160 | 4f | NS3 and NS5B |
| NC_009826 | 5a | NS3 |
| EUH1480 | 5a | NS5B |
| DQ278892 | 6 | NS3 and NS5B |
| AY859526 | 6a | NS3 and NS5B |
| NC_009827 | 6b | NS3 and NS5B |
| EF108306 | 7a | NS3 and NS5B |
